# The design of macromolecular crystallography diffraction experiments

**DOI:** 10.1107/S0907444911007608

**Published:** 2011-03-18

**Authors:** Gwyndaf Evans, Danny Axford, Robin L. Owen

**Affiliations:** a Diamond Light Source, Harwell Science and Innovation Campus, Didcot OX11 0DE, England

**Keywords:** macromolecular crystallography, microcrystallography, X-ray beamlines, synchrotron radiation

## Abstract

The measurement of X-ray diffraction data from macro­molecular crystals for the purpose of structure determination is the convergence of two processes: the preparation of diffraction-quality crystal samples on the one hand and the construction and optimization of an X-ray beamline and end station on the other. Like sample preparation, a macromolecular crystallography beamline is geared to obtaining the best possible diffraction measurements from crystals provided by the synchrotron user. This paper describes the thoughts behind an experiment that fully exploits both the sample and the beamline and how these map into everyday decisions that users can and should make when visiting a beamline with their most precious crystals.

## Abstract 2

Thoughts about the decisions made in designing macromolecular X-ray crystallography experiments at synchrotron beamlines are presented.

## 1. Introduction

The measurement of X-ray diffraction data from macromolecular crystals is considered to be a routine practice and has led to the determination of tens of thousands of atomic structures. The introduction and ongoing development of semi-automated programs suggesting the optimal diffraction experiment at most synchrotron sources (Leslie *et al.*, 2002 ▶; Bourenkov & Popov, 2010 ▶) has sought to make the collection of good-quality X-ray data possible for the non-expert user. However, it is still the case that many of the decisions that determine the quality of diffraction data must still be made manually at the time of the experiment and are neither automated nor easily automatable. This is especially true in cases where samples diffract poorly. Data quality can sometimes be improved by changing beamline parameters such as the X-ray beam size or the X-ray focal length, but such decisions are not easy to automate. Furthermore, some of these decisions are not straightforward and require a good understanding of X-ray beamlines and measurement science.

If high-quality data are to be collected, then the goal of the experiment and the criteria used to judge success must first be defined. Here, we briefly review the MX experiments most commonly carried out at synchrotron sources and the metrics used to judge data quality. We also summarize considerations for the setup of beamline equipment for the collection of optimal diffraction data and describe how the effective use of a beamline can improve data quality.

## 2. Defining the diffraction experiment and assessing its success

In order to carry out the optimal diffraction experiment given the crystals at hand, the desired goal should first be well defined. Measurements routinely made by macromolecular crystallographers have one or more of several aims, which are summarized in the following sections.

### 2.1. Classes of experiment

#### 2.1.1. Sample characterization

This encompasses early-stage characterization of crystallization conditions with a view to optimization, assessment of the unit-cell parameters, Laue-group determination, diffraction strength and resolution range for comparison against other crystal forms, evaluation of the radiation-sensitivity of a crystal, the location of a small crystal within its sample mount and the location of a well diffracting subvolume in a larger inhomogeneous crystal. Sample characterization is a critical part of any diffraction experiment or indeed any crystallographic structure determination, as it provides vital knowledge about how one should proceed with diffraction measurements or whether effort would be better spent on further sample preparation. Generally, complete data are not required for sample characterization and in many cases one or two diffraction images may provide sufficient information, although more images may be required for unambiguous Laue-group determination.

#### 2.1.2. Experimental phasing or structure solution

The type of measurements required for the determination of a structure will depend on the structure-solution method being employed, whether SAD, MAD or molecular replacement (MR). Strategies for the optimal collection of anomalous data and determination of the required rotation range are explained in detail elsewhere (Dauter, 1999 ▶, 2010 ▶). In all cases, however, a complete high-quality data set is preferred. In most cases the resolution of the data used for initial phase determination can be relatively modest (∼3 Å) and it is preferable, especially for MAD and SAD, to collect complete data to a lower resolution than to overexpose the crystal causing deterioration in diffraction and possibly leading to incomplete or inferior data. In cases where the anomalous signal is weak, for example where the signal from native S atoms is being used, the measurement of very high multiplicity data has been found to be invaluable in producing data of sufficient accuracy to permit structure solution (Wang *et al.*, 2006 ▶), but the effects of radiation damage must again be considered carefully and a compromise on the resolution of measured data for phasing must usually be made (Debreczeni *et al.*, 2003 ▶). A high-resolution data set for phase extension and refinement may be measured separately where high multiplicity is not a requirement.

#### 2.1.3. Resolution extension

If an atomic structure has already been determined but to lower resolution than desired, diffraction data may be recorded to higher resolution for the purposes of improving model accuracy. Complete data are necessary but no additional experimental phase information is being sought.

#### 2.1.4. Ligand identification

The detection of ligands bound to structures is the main activity of synchrotron users from the pharmaceutical industry (Skarzynski & Thorpe, 2006 ▶). Usually, only modest resolution (2.5 Å or better) data are required to identify binding without ambiguity, but completeness is essential in order to avoid electron-density artefacts that might be misinterpreted.

### 2.2. Criteria for assessing success

Any experiment is carried out to answer questions posed by researchers. Good data are therefore those which provide sufficient information to allow the scientific question to be answered or can strongly support a given conclusion. What is ‘good for the goose’ may not necessarily be ‘good for the gander’; that is, a data set needed to provide atomic detail around an active site may not be the same as a data set needed to provide accurate low-resolution experimental phase information. The definition of good data is itself not clear-cut, but it would be generally accepted (Kleywegt, 2000 ▶) that the following factors dictate data quality to a greater or lesser extent. A series of quality indicators are available and have been described elsewhere (Evans, 2006 ▶) but are briefly reviewed here. It should be noted here that the speed and high quality of modern data-analysis and structure-solution software (Minor *et al.*, 2006 ▶; Vonrhein *et al.*, 2007 ▶; Pape & Schneider, 2004 ▶; Terwilliger & Berendzen, 1999 ▶) means that one major indicator of data quality is whether the structure can be solved or whether the desired result can be automatically achieved.

#### 2.2.1. Signal-to-noise ratio

No single number can be suggested as an acceptable signal-to-noise ratio (SNR) because it depends on the requirements of the experiment. For example, if one is attempting to detect a very weak anomalous scattering signal [for example, in a sulfur single-wavelength anomalous diffraction (S-SAD) experiment] for the purposes of substructure determination and phasing the SNR needed would be higher than that required if the data were to be used for the refinement of an already solved structure. One usually refers to the overall SNR of the data and the SNR in the highest resolution shell as an indicator of quality. Conversely, the high-resolution cutoff is usually determined by an SNR threshold, typically 2 > *I*/σ(*I*) > 1, *i.e.* approaching the threshold where on average noise begins to be larger than the measured signal.

#### 2.2.2. Completeness and redundancy/multiplicity

Data should be as complete as possible, *i.e.* accurate measurements of *I*(*hkl*) for all *hkl* (or symmetry-equivalent indices) within the desired resolution range. In particular, there is an expectation that the completeness should be close to 100% across the full resolution range. Measured intensities should have sufficient SNR and not be overloaded (above the maximum count threshold of the detector). If anisotropic anomalous scattering (AAS), which locally breaks the symmetry around heavy-atom sites, is to be accounted for in data analysis (Schiltz & Bricogne, 2010 ▶), then in the absence of any knowledge of the local heavy-atom environment the completeness should be assessed assuming *P*1 crystal symmetry since unmerged data are used for AAS analysis.

#### 2.2.3. Merging statistics

Merging-quality indicators for diffraction data after the scaling and merging step have been described in detail (Diederichs & Karplus, 1997 ▶; Weiss, 2001 ▶; Weiss & Hilgenfeld, 1997 ▶) and are by far the most heavily utilized indicators by crystallographers. These indicators quantitatively describe the internal consistency of the data and indicate the level of random error present while also representing the residual systematic error in the data, although the two are difficult to separate. These indicators are often weighted by the number of contributing measurements, *e.g. R*_meas_ or *R*_r.i.m._ (multiplicity-weighted *R*_merge_) or *R*_p.i.m._ (the precision-indicating *R* factor). Other useful values are the internal correlation coefficient taken between two randomly selected half data sets calculated between measured intensities, *I*, and between derived anomalous differences, Δ*I*, where the presence of anomalous signal is expected. This anomalous correlation coefficient determined as a function of resolution can be a powerful indicator of the useful resolution range of anomalous data to be used for anomalous scattering substructure determination (Debreczeni *et al.*, 2003 ▶). Furthermore, the anomalous correlation coefficient between measurements at different wavelengths in the case of multiple-wavelength anomalous diffraction (MAD) can be a strong indicator of the quality of MAD data and the resolution to which the anomalous signal is useful (Schneider & Sheldrick, 2002 ▶).

### 2.3. Resolution range

The resolution range of a good data set is the range within which the data can be said to be essentially complete. Typical good practice would require the low-resolution data to extend down to at least 30 Å *d*-spacing, but this would be even lower for larger molecules within larger unit cells. Accurately measured low-resolution data are particularly useful for assisting solvent-flattening and molecular-replacement methods, in which definition of the molecular envelope (principally defined by low-resolution structure factors) is important. The high-resolution limit for a data set (defined by the SNR cutoff) is determined by either the diffracting limit of the crystal, which is in turn related to its intrinsic atomic and molecular order, or by parameters controlling data collection such as exposure time or incident flux. Measurement of higher resolution data will generally require a greater X-ray dose to be delivered to the crystal, leading to increased radiation damage. It is important that an experimenter knows what resolution is required in order to answer the scientific question at hand. Needlessly overexposing a crystal can, through the onset of radiation damage, lead to incomplete data at the desired resolution and/or specific structural changes during data collection.

### 2.4. Radiation damage

An awareness of radiation damage and its consequences (Garman, 2010 ▶) is essential when performing measurements on modern third-generation synchrotron beamlines with small crystals (<50 × 50 × 50 µm). Ideally, a data set free of global and site-specific radiation damage is desirable, but may not always be achievable. The goal of the experiment will dictate how much damage is tolerable. For example, in heavy-atom phasing, where the occupancy of heavy atoms is proportional to the measured anomalous signal, minimizing heavy-atom site-specific damage is important for the success of phasing (Holton, 2007 ▶). Similarly, where the chemical details of an active site are critical for an understanding of enzymatic function, the avoidance of radiation damage at the site is a pre­requisite. An added complication is that X-ray-induced damage may occur very rapidly at the active site (see, for example, Yano *et al.*, 2005 ▶) and can be essentially complete before any appreciable change in global parameters such as diffracting power is observed. A careful experimental strategy is essential and confirmation of the active-site state by a complementary method such as UV–Vis absorption spectroscopy (Beitlich *et al.*, 2007 ▶ and references therein) or XANES (Yano *et al.*, 2005 ▶) may be necessary.

Programs are available that can assist in assessing the expected level of damage and then designing measurement strategies around this. These are, respectively, *RADDOSE* (Paithankar & Garman, 2010 ▶) and *BEST* (Bourenkov & Popov, 2010 ▶). Very recently, it has been observed that site-specific radiation damage can be drastically reduced at temperatures below 100 K, with a slight reduction in global damage also being seen (Meents *et al.*, 2010 ▶). This discovery might permit better preservation of structural details during data collection from metalloproteins, where radiation damage has previously been problematic, and might also enable better anomalous data to be measured owing to better preserved integrity of the anomalously scattering substructure through­out data collection.

## 3. Instrument design and tools for data collection

Key to the success of structural biology by X-ray crystallo­graphy has been the provision of X-ray instrumentation that permits the easy and efficient collection of high-quality diffraction data from users’ samples (Cassetta *et al.*, 1999 ▶). The high-throughput capability of X-ray beamlines has been a focus of attention in the last 10–15 years, driven partially by structural genomics initiatives in Europe, the USA and Japan (Edwards, 2009 ▶; Fogg *et al.*, 2006 ▶; Yokoyama *et al.*, 2000 ▶; Joachimiak, 2009 ▶). However, high levels of automation are also being used to progress very challenging projects by allowing crystallo­graphers to focus on important scientific details whilst lifting the tiresome burden of repetitive tasks such as the manual mounting of samples in the beamline and centring of samples in the X-ray beam. However, even with all these automation tools in place the success or otherwise of a diffraction experiment can still hinge on simple decisions related to aspects of instrumentation that remain under the control of users and beamline staff. The following sections describe the impact of beamline design on the experiment and describe some good practice that can improve the final results obtained from the experiment.

### 3.1. Relationship between beamline and sample

Beamline design is driven by the characteristics of the crystal samples that are to be measured and by the practical needs of the crystallographer who uses the beamline. The sample characteristics that principally influence data quality and hence the design of an experiment have been described elsewhere (Nave, 1999 ▶) and can be briefly summarized as  follows: unit-cell length dimensions, mosaicity or expected crystal internal order, diffraction strength (related to molecular weight, solvent content and unit-cell volume), crystal size and shape, sample-mounting method, sample-cooling method and radiation-sensitivity.

The range of expected values of each parameter directly impacts the beamline in the following ways. The upper limit of resolvable unit-cell length is influenced by the X-ray beamsize at the detector, the beam divergence, the detector point-spread function (PSF), the maximum sample-to-detector distance and, indirectly, the detector size, since this will impact on the highest resolution of diffraction measurable at the largest crystal-to-detector distance.

In order to record optimal data from crystals with a high mosaic spread, or crystals of low internal order, the minimum X-ray beam size at the sample and the maximum beam divergence become relevant. Furthermore, the angular precision and repeatability of the sample-rotation stage must be sufficient to finely sample crystal reflections with a high degree of synchronization with the X-ray shutter and the detector. Crystal order also has a considerable impact on X-ray optics and end-station design since it defines an acceptable frequency range for both positional and intensity vibrations in the X-ray beam. Somewhat counterintuitively, the better ordered a crystal the greater the demand placed upon the beamline optics quality. Mechanical vibrations present in the beamline and source become more apparent, especially at high data-collection rates, since the crystal itself performs less angular- and time-averaging the lower its mosaicity.

The diffraction strength and the dynamic range of diffraction patterns define the maximum X-ray flux required by a beamline and also set the requirements for the detector dynamic range and the acceptable level of inherent detector noise. The typically weak diffraction from macromolecular crystals with high average *B* factors implies that the observed diffraction will span several decades in intensities and will require, on average, very intense beams to measure it.

The crystal size and shape map directly onto requirements for beam-size range and beam-shape range, while also influencing the choice of detector parameters such as the PSF. Recent years have seen the need for X-ray beams of a few micrometres in size that help address the problems of growing large diffracting crystals (Perrakis *et al.*, 1999 ▶; Riekel *et al.*, 2005 ▶).

The methods and process of sample mounting drive decisions about the sample environment such as sample-visualization methods, sample support and the positioning of key equipment such as the cryogenic gas stream and the fluorescence detector. Similarly, the typical radiation-sensitivity of samples at room temperature or when cryocooled to 100 K or less will affect the minimum exposure time, shutter properties, sample environment and automated sample-mounting robotics. Moreover, the quality of the sample mount can have a direct bearing on data quality and attention should be paid to this by the user (Alkire *et al.*, 2008 ▶; Flot *et al.*, 2006 ▶).

Certain instrumental factors can be detrimental to data quality and should be minimized or removed by design. X-ray scatter from the instrument, principally from defining apertures, air or the cryocooling gas stream, creates background in the diffraction image and reduces the SNR. Errors in the timing of the opening and closing of the X-ray shutter with respect to the crystal rotation can introduce errors in the relative intensities of symmetry-equivalent partially (*I*_part_) and fully (*I*_full_) recorded reflections, leading to, on average, *I*_part_ > *I*_full_. Similarly, errors in the angular velocity of rotation of the sample data-collection axis can map into uncertainties in the scale factors of diffraction intensities. Instability in end-station apparatus, sample mounting and/or errors in the beam intensity and position at the sample will also affect the measured intensities. Finally, instrument-calibration errors such as an incorrect X-ray wavelength or detector distance can create problems in data analysis and sometimes reduce expected signals in anomalous scattering experiments. X-­ray detection-related errors such as a poorly calibrated detector will globally affect data in a systematic way.

For a beamline to operate successfully and provide the best data from a sample, each of these potential sources of error must be carefully dealt with through design, construction, commissioning and then careful maintenance and operation of the instrument.

**Figure 1:**
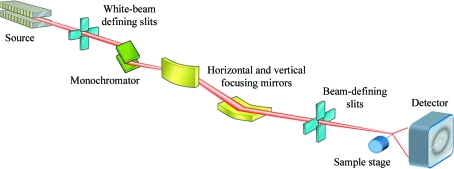
Simple schematic diagram showing the major components of a typical MX beamline. The source is typically a bending magnet or insertion device. Moving downstream, this is followed by a monochromator for selecting X-rays of a single wavelength, focusing optics and beam-defining slits, which together define the beam size and shape at the sample.

A schematic diagram of a typical MX beamline is shown in Fig. 1 ▶. The key elements of a beamline, a monochromator for selecting X-ray wavelength, mirrors for the focusing and shaping of the X-ray beam, slits or fixed apertures of various sizes for cleaning and further shaping of the beam and the sample goniometer and detector for performing the measurement using the rotation method are all shown. See Helliwell (1992 ▶) for a comprehensive description of X-ray instrumentation for MX.

### 3.2. Choice of wavelength

The monochromator defines the energy, or wavelength, of the X-rays that are incident on the sample. At the majority of tuneable MX beamlines an Si(111) double-crystal monochromator is used, providing a measured energy resolution at the sample of typically ∼2 × 10^−4^ (Δ*E*/*E*).

A number of studies have been performed to investigate the effect of X-ray wavelength choice on data quality or structure determination. Studies by González (2003*a* ▶,*b* ▶) have focused on strategies and optimal wavelength choices for multi-wavelength experiments utilizing anomalous diffraction. The studies concluded that where a suitable absorption edge is accessible by a beamline, single-wavelength SAD (measured at the *f*′′ maximum) or two-wavelength MAD data (at *f*′ minimum and at a remote wavelength to maximize Δ*f*′) measurements are optimal and three-wavelength measurements provide little in terms of additional phase information for structure determination and risk the concern of additional radiation damage.

The use of softer X-rays for SAD structure determination has been investigated with a view to finding the optimal wavelength for such measurements (Mueller-Dieckmann *et al.*, 2005 ▶). The authors found that data measured using 2.1 Å X-­rays routinely produced the best anomalous signal-to-noise ratio and that this was virtually independent of the anomalous scattering substructure. This was in the absence of any nearby absorption edges. The authors were careful to recommend that a longer wavelength data set should be accompanied by a short-wavelength data set that might increase the measured resolution range if required.

In the absence of an anomalous scatterer with accessible absorption edges, the diffraction intensity for a given dose absorbed in the sample, *I*_E_, is approximately constant over the energies commonly used in MX (Arndt, 1984 ▶). While *I*_E_ increases by a small fraction at higher X-ray energies, the effect for most crystal sizes is small and a decline in other factors such as the detector efficiency will in all likelihood negate any gain. This implies that wavelength selection has little effect on one’s ability to measure complete data before radiation damage sets in where absorption edges are not present. The choice of wavelength is therefore mainly dictated by the presence of anomalous scatterer absorption edges, the desired resolution of the experiment, the avoidance of sample- or air-absorption effects and detector-sensitivity considerations.

The accessible wavelength range of a beamline and the available photon flux across this range may of course be a limiting factor for some experiments, making it an important consideration in the user’s choice of beamline.

### 3.3. Matching beam size to sample size

Focusing at MX beamlines is achieved by the use of mirrors [most commonly either Kirkpatrick–Baez (KB) or toroidal] or a sagittal bender. In some cases optimization of the mirrors may not be possible by the user or it may be optimal to define the beamsize at the sample by slits.

**Figure 2:**
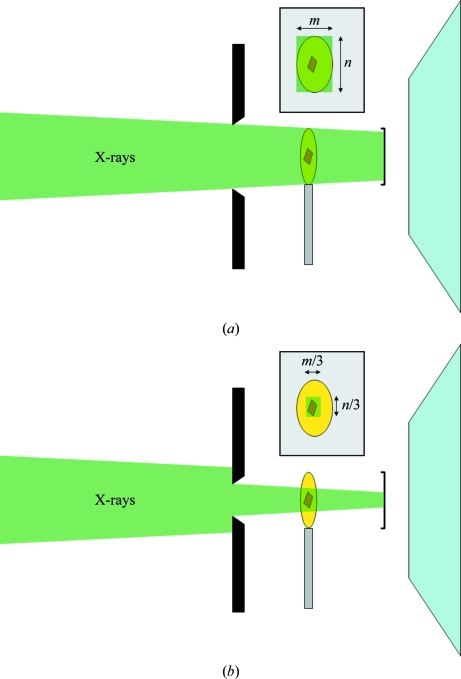
Schematic diagram showing how careful use of defining slits before the sample position can help to match the X-ray beam size to that of the crystal sample. This can significantly reduce the amount of undesirable scattered X-rays generated by interaction with noncrystalline material surrounding the crystal.

In optimizing the signal-to-noise ratio in macromolecular crystallography experiments one should principally focus on reducing the background. Increasing the signal by exposing for longer brings with it undesirable consequences such as an increased likelihood of radiation damage. Reducing the X-ray background in an experiment may indeed improve the signal-to-noise ratio to such an extent that the exposure time can be reduced further, mitigating radiation-damage effects. On many beamlines an improved signal-to-noise ratio can be achieved by trimming the X-ray beam shape using defining slits positioned just upstream of the sample, as illustrated in Fig. 2 ▶. Reducing the volume of nondiffracting material that X-­rays impinge on is an essential part of X-ray data collection.

**Figure 3:**
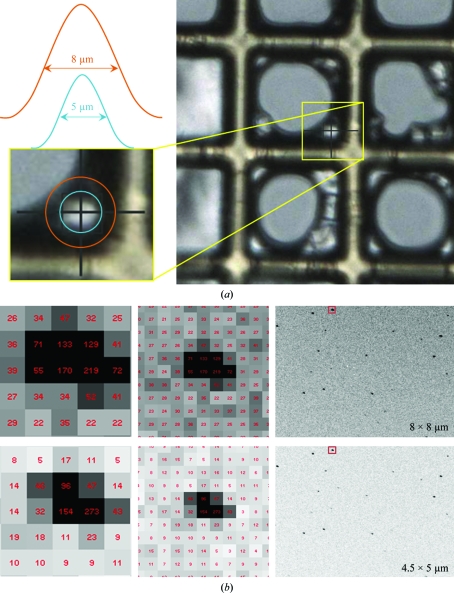
X-ray background reduction obtained by precisely matching X-ray beam and crystal size. (*a*) AcMNPV polyhedra crystals of approximate dimensions 5 × 5 × 5 µm were mounted on a Micromesh grid. The enlarged area illustrates the FWHM extent of the two different beam sizes used for this test aligned around one of the polyhedra crystals. The Gaussian profiles shown are for illustration purposes only. (*b*) Identical regions of diffraction images from the crystal in (*a*) taken using an 8 × 8 µm beam for 1 s and a 4.5 × 5 µm beam for 3.3 s. Exposure times were adjusted so that the average integrated intensity from both images was similar. The images were displayed with *ADXV* using identical contrast levels. The inserts show pixel values in the region of the same diffraction spot (−12 −3 17), where a reduction in average background of about 1/3 is observed. This reflection was chosen because it was a fully recorded reflection of significant intensity as indicated by *MOSFLM*.

For microbeams and microcrystals this message is paramount and its importance can be illustrated by a simple example of diffraction from 5 × 5 × 5 µm protein crystals obtained using different X-ray beam sizes. Two beam sizes were used: a standard setting of 8.0 × 8.0 µm with 10^12^ photons s^−1^ and a setting of 4.5 × 5.0 µm achieved in this case by reducing the secondary-source size of I24 at the expense of a 14.3-fold reduction in overall flux as measured by a PIN diode at the sample position. The sample crystal, polyhedrin of the baculovirus *Autographa californica* multiple nucleopolyhedrovirus (AcMNPV; Ji *et al.*, 2010 ▶), is shown in Fig. 3 ▶(*a*). Diffraction images (regions of which are shown in Fig. 3 ▶ *b*) were measured on the I24 microfocus beamline (Evans *et al.*, 2007 ▶) at the Diamond Light Source using a Pilatus 6M detector (DECTRIS Ltd, Baden, Switzerland). Table 1 ▶ shows the characteristics of the two beams used and the results of the integration of each image using *MOSFLM* (Leslie, 2006 ▶). A significant decrease in X-ray background across the whole detector area is achieved by reducing the FWHM beam size from being mismatched in size at 8.0 × 8.0 µm to being well matched at 4.5 × 5.0 µm. Fig. 3 ▶(*a*) illustrates how the extent of the approximately Gaussian profile of the beam compares with the crystal. The exposure times for both images were chosen so that the average integrated intensities of fully recorded reflections, 〈*I*〉, from *MOSFLM* were similar for both images. This resulted in exposure times of 1 and 3.3 s, respectively. Further experimental details are given in the legend of Table 1 ▶. Fig. 3 ▶(*b*) shows the clear threefold reduction in X-ray background using the smaller beamsize and this was reflected in a concomitant increase in the measured average signal-to-noise ratio 〈*I*/σ(*I*)〉 of the data. It was observed for the matched beam that 〈*I*/σ(*I*)〉 = 1.5 in the highest resolution bin to 2.5 Å. For the larger mismatched beam an equivalent 〈*I*/σ(*I*)〉 of 1.6 was observed in the resolution shell extending to 2.89 Å [the 〈*I*/σ(*I*)〉 at 2.5 Å was 0.4]. The potential for extending the useful resolution of diffraction data by a simple reduction in beamsize (even at the expense of X-ray flux) is clear in this example.

It is worthwhile mentioning here that at the level of a few micrometres vibrations of the crystal sample owing to the flow of cold nitrogen gas over it may become significant and could actually lead to deterioration of data quality owing to the sample moving in and out of the similarly small beam. It is therefore important that users and beamline scientists are cautious at every stage of sample and beamline preparation in order to prevent the introduction of such errors.

A similar argument extends to the measurement of data from crystals with a plate-like morphology, where the projected size of the crystal, as seen by the X-ray beam, changes greatly as the crystal rotates. The benefits of measuring data from a plate-shaped crystal using different beam sizes can be illustrated by an example (Hausmann *et al.*, 2010 ▶) using a crystal of a glycoprotein autotaxin/ENPP2 measured on the Diamond I24 microfocus beamline. The crystal was approximately 200 × 30 × 1 µm in size and data were measured using three different beam sizes, 8 × 8, 15 × 20 and 30 × 50 µm, each delivering the same photon flux of ∼10^12^ photons s^−1^. Hausmann and coworkers were able to record complete data using Diamond I24 from a single plate-shaped crystal, whereas previously they had been required to merge data from two different crystals recorded on Swiss Light Source beamline X06SA using both the high-resolution (beam size 40 × 100 µm) and microcrystal diffractometer (beam size 10 × 10 µm) setups. The problem was the lack of complete data measured using the 10 × 10 µm beam when the crystal was oriented face-on to the beam. The advantage of the I24 variable beam for measuring complete data from the plate crystal can be explained as follows.

The small beam size was used to measure data when the crystal was ‘edge-on’ to the beam and larger beams were used when the projected crystal size increased with rotation. Using a small focal spot for the edge-on orientation ensured that as much flux as possible was incident on the crystal and that minimal background scatter was measured. Use of a larger beam for the face-on orientations distributed the high flux across a larger volume, thereby reducing the absorbed dose and the potential for radiation damage. These arguments can be considered quantitatively using an idealized example. Consider a 5 × 100 × 100 µm crystal and two possible beam sizes 5 × 5 µm and 25 × 25 µm both having identical flux. Edge-on the small beam intersects a crystal volume of 2500 µm^3^, whereas face-on a small beam would intersect a volume of only 125 µm^3^, thereby requiring a 20-fold longer exposure time to achieve the same average intensity per image. Furthermore, the deposited dose per unit volume is increased by a factor of 20. Using a large beam for the face-on data collection increases the illuminated volume to 3125 µm^3^ and therefore roughly equivalent average intensities would be achieved with 4/5 of the exposure time. More importantly, the deposited dose would be distributed into a larger crystal volume, thereby slowing the onset of radiation damage.

Using an X-ray beam size that is smaller than the crystal is to be avoided unless the crystal is inhomogeneous (see §[Sec sec3.4]3.4) or unless employing a special strategy such as helical scanning across needle-shaped crystals (Flot *et al.*, 2010 ▶). It has been demonstrated that for a well diffracting and homogeneous sample the use of a beam size comparable to that of the crystal provides the best-quality data (Sanishvili *et al.*, 2008 ▶).

### 3.4. Characterization of inhomogeneous crystals

**Figure 4:**
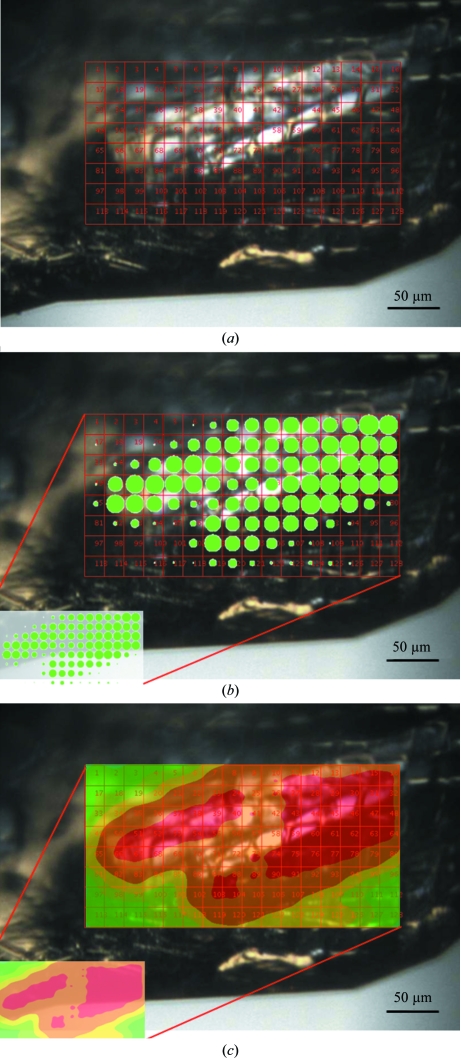
Results of grid scans measured using crystal(s) of thermolysin on beamline I24 at Diamond. The grid (*a*) defines the area over which the scan will be made and the distance between successive measurements. The images were analysed by *DISTL* (Zhang *et al.*, 2006 ▶) and results indicating diffraction strength and/or quality are displayed as filled circles (*b*), the radii of which are indicative of the score. In this case scores based on the number of Bragg candidates are displayed, but other *DISTL* outputs, such as total diffracted signal or resolution limit, can be used. As an alternative to the filled circles a contour plot (*c*) can be displayed which may aid the identification of crystal edges and subdomains.

Many crystals, especially those of multi-protein complexes and membrane proteins, will naturally tend to form inhomogeneous crystals where the diffraction quality varies significantly throughout the crystal volume. Illuminating the whole crystal with the X-ray beam can under these circumstances give poor-quality diffraction characterized overall by high mosaicity, poor spot shape and limited resolution. Experience on several microfocus beamlines has shown that for inhomogeneous crystals using a beamsize considerably smaller than the crystal size can sometimes greatly improve diffraction quality because a small well ordered region can be located and preferentially illuminated. The success of this method relies on the availability of tools that enable the well diffracting sub­volume to be easily found. Typically, a crystal is scanned through the beam and a diffraction image is recorded at each location on the crystal on a two-dimensional grid. The diffraction patterns recorded at each position are then analyzed and quantified in terms of their quality (see Fig. 4 ▶). Two recent examples of this are the grid-scanning tools provided at all MX beamlines at the Diamond Light Source (Aishima *et al.*, 2010 ▶) and crystal cartography developed at the ESRF (Bowler *et al.*, 2010 ▶). The former was developed at the I24 microfocus beamline to enable the straightforward location and characterization of crystals. The I24 grid-scanning tool utilizes the high-speed readout of the Pilatus 6M detector to perform continuous scans across a sample while recording the diffraction data at any given point. The scans can be performed very quickly and the results are displayed overlaid on the crystal sample in either the form of ‘blobs’ or as a contour plot. An example of this is given in Fig. 4 ▶, where a grid scan has been performed on a thermolysin sample using a 20 × 20 µm beam.

### 3.5. Choice of attenuation/exposure time

The degree of attenuation and the exposure time are, of course, closely linked. Several studies have shown that global radiation damage appears to be independent of the rate at which dose is deposited in the crystal and only depends on the total absorbed dose (see Garman, 2010 ▶, and references therein). Determining the optimal dose per image depends largely on the goal of the experiment (§[Sec sec2.1]2.1), the diffracting power of the crystal (§[Sec sec2.2.1]2.2.1) and the radiation-sensitivity of the system under study. The diffracting power can be established by integrating a small number of test images and the radiation-sensitivity can be determined by collecting a high-dose data set from a sacrificial crystal and determining the rate of decay. The relevance of this sensitivity estimate to other crystals does of course depend on the homogeneity of the samples. Alternatively, an estimate of the crystal lifetime can be computationally obtained from the unit-cell contents and beamline parameters using the program *RADDOSE* (Murray *et al.*, 2004 ▶). The dose absorbed either per image or over the duration of a data set can then be compared with established dose limits.

Instrumental factors such as the error in the time taken to open or close the shutter or the dynamic range of the detector should also be taken into account. Long exposure times may result in overloads for low-resolution reflections, whilst for very short exposure times (of the order of milliseconds) uncertainties in shutter-opening times and synchronization of beamline components may affect data quality. Shutter-timing questions are of less relevance when using continuous-readout detectors such as pixel-array detectors (PADs).

### 3.6. Detector considerations

The crystal-to-detector distance (XTD) is one of the most commonly changed experimental variables and determines the resolution range over which diffraction data are recorded (see §[Sec sec2.3]2.3). Decreasing the XTD allows data to be collected to a higher resolution at the expense of an increased contribution of incoherently scattered radiation to the recorded image. The XTD should therefore be carefully set, as improved data can be collected by increasing the XTD so that the inscribed circle matches the resolution required from the experiment.

The type of detector should also be factored into the experimental strategy. Fast-readout PADs allow more than ten frames per second to be collected and improved data can be obtained by using a fine ϕ-slicing data strategy with no con­comitant time penalty. Fine ϕ-sliced images will, in general, result in fewer spatial overlaps and fewer pixels that are saturated or require a count-rate correction (Pflugrath, 1999 ▶). For fine ϕ-sliced data the time (or dose) per degree should be kept constant, *i.e.* a single 1° oscillation 1 s image would become ten 0.1° 0.1 s images. Shutter problems such as those alluded to in §[Sec sec3.1]3.1 are circumvented by continually reading out the detector with the shutter remaining open for the entire duration of the data set (Brönnimann *et al.*, 2003 ▶). The pixel size should also be taken into account, as large unit cells will result in closely spaced spots which need to be resolvable. For problematic or low-quality data sets an accurate beam centre is essential. This can be confirmed by the collection of a highly attenuated direct-beam image or alternatively the beam centre and other beamline parameters can be cross-checked by the collection of data from a well diffracting test crystal. Collection of data from a well diffracting test crystal is often preferable, as reliably refined experimental parameters can then be used to process weak or troublesome data.

For fine ϕ-sliced data collection the optimum rotation range of each image is typically a function of the crystal mosaicity and detector type. A detailed review of fine ϕ-slice data collection is given by Pflugrath (1999 ▶).

## 4. Discussion

The collection of diffraction data from macromolecular crystals is a well established experiment with, in many cases, well defined and partially automated protocols. Such protocols may suggest the optimal position on a crystal from which to collect data or the optimal angular range over which data should be collected. In many cases, however, two input variables are missing from the creation of an experimental strategy and significantly improved data can be collected by taking these into account. Firstly, the goal of the experiment and acceptable criteria for success should be defined. Secondly, the full range of variable beamline parameters such as the beamsize at the sample, beam divergence and energy should be exploited. Spending more time considering and optimizing these parameters can often be an excellent use of synchrotron beamtime, resulting in better quality data for use in the subsequent steps of structure determination. Time spent thinking and planning at the beamline may avoid the need for future trips to a beamline in order to re-measure data. Finally, it is important to mention that it is very difficult to recover from a poorly designed experiment, poorly measured data or data that have been measured on an inferior or faulty instrument. The importance of having experienced beamline staff supporting users and maintaining the X-ray facilities cannot be underestimated in this respect and the key to successful experiments lies in continued dialogue and collaboration between users and staff.

## Figures and Tables

**Table 1:** Data-collection settings and spot statistics for the two images measured using matched and mismatched beam sizes The exposure time of 3.3 s for the matched beam settings was determined from the average integrated intensity, 〈*I*〉, of a previous single 1 s exposure with the same beamsize. Notice that a 14.3-fold reduction in overall flux was experienced with the smaller beam, but the exposure time was only increased by 3.3-fold to achieve equivalent 〈*I*〉 for both matched and mismatched images. Only fully recorded reflections extending to 2.5 Å resolution were considered for the analysis. Although the improvement in 〈*I*/σ(*I*)〉 from 2.7 to 3.7 may not appear to be dramatic, the potential increase in resolution of such a background reduction is more significant. The resolution shell where 〈*I*/σ(*I*)〉 ≃ 1.5 extends to only 2.89 Å using the mismatched beam, whereas for a matched beam it extends to 2.5 Å.

Beam size (µm)	Flux (photons s^−1^)	Experiment time (s)	〈*I*〉	〈*I*/σ(*I*)〉	Resolution range where 〈*I*/σ(*I*)〉 ≃ 1.5 (Å)
8.0 × 8.0	1 × 10^12^	1.0	77	2.7	3.16–2.89
4.5 × 5.0	7 × 10^10^	3.3	76	3.7	2.67–2.50
